# A structured remediation program for communication skills

**DOI:** 10.5116/ijme.5d5a.72c3

**Published:** 2019-08-29

**Authors:** Pedro Morgado, Ana R. Lemos, Sara Almeida, Joao J. Cerqueira, Nuno Sousa

**Affiliations:** 1 Life and Health Sciences Research Institute (ICVS), School of Medicine, University of Minho, Braga, Portugal; 2 ICVS/3B's, PT Government Associate Laboratory, 4710-057 Braga/Guimarães, Portugal

**Keywords:** Structure, remediation program, communication skills, Communication Assessment Scale score, Portugal

The doctor’s ability to communicate effectively is critical both to clinical practice and to health outcomes.^1^ Indeed, effective patient-doctor communication has been extensively shown to improve patient satisfaction and patient health outcomes.^2^ Focus on acquisition and training of these skills is now given in medical school curricula. As with every other skill, performance in communication tasks is variable between individuals. There is much written on teaching and assessment^3^^,^^4^ but the remediation of students’ communication skills is rarely addressed in the literature. To our knowledge, there are no standardized interventions to address communication deficiencies, which boosts the need for designing and reporting that type of interventions. In this report, we present and discuss our experience of developing and implementing a standardized remediation program in clinical interview communication skills for third-year medical students studying in a 6-year medical curriculum at the University of Minho.

All students that failed in the clinical skills examination at the end of the third year were invited to join the program. More specifically, these students failed the history taking and/or communication components on the high-stakes Objective Structured Clinical Examination (OSCE), backed by a solid standardized patients (SP) program, specifically designed to assess the mastery of history-taking and physical exam skills just before the start of their clinical rotations.^5^ The exam included six stations of 15-minutes SP encounters, each station with a clinical history and specific physical examination. Performances were scored using checklists by clinical monitors (junior and senior doctors) – on history taking, physical examination and communication skills - and by trained SPs – on communication skills only. History and physical examination checklists were custom made according to the patient scenario while communication was always assessed with the same Communication Assessment Scale (CAS).

The aims of the program were: (i) to improve students’ awareness of their difficulties and to stimulate students to seek active strategies to deal with those problems; (ii) to stimulate students to engage in extra-curricular activities that can enhance their communication skills; (iii) to train communication competences in the context of a clinical interview with standardized patients (SP); (iv) to remediate students that failed in the clinical skills examination at the end of the third year. A total of 6 students were enrolled in the program that is composed of 7 steps.

In step 1 (identifying communication problems), each student takes a clinical history from an SP. Right after the clinical encounter, each student makes an informal self-assessment on his/her performance and receives feedback from the clinical monitor and the SP. In this step, students re-assess communication abilities and receive structured feedback.

During step 2 (self-assessment videotape review), each student takes a clinical history from an SP (that is videotaped) and reviews his/her performance by analyzing the video. The student proposes (and receives suggestions of) strategies to improve his/her communications skills (e.g. study the clinical semiology to improve medical knowledge, read literature books to improve language skills, engage a theatre group to train posture, perform informal role plays with colleagues). In this step, students increase their awareness of their communication insufficiencies and personalized interventions could be drawn.

In step 3 (building a schema/checklist for each clinical presentation) occurs at least one month after the previous. In this step, all students elaborate schemas and checklists on different clinical presentations of disease to improve their clinical reasoning and their awareness of critical questions/subjects to approach in each clinical scenario. Each student shares his/her own checklists with the other participants in the program. In this session, students improve their ability to elaborate on differential diagnosis and elucidate obtained information.

In steps 4 and 5 (SP encounters) students collect clinical histories from SPs. To prepare students for these encounters, information about the major complaint is provided the day before each encounter. After the clinical interview students do a self-assessment of their performance and receive feedback from the clinical tutor and the SP. They also receive information on history checklist score and on Communication Assessment Scale (CAS) score.

In step 6 (Group videotape review), each student collects a clinical history from an SP and reviews his/her performance based on the video analysis. Additionally, another student from the group is selected to comment on the performance of that colleague. In this session, students notice their improvements in communication and interviewing skills and train to provide feedback to others and, as a consequence, to enhance their critical view on communication competences.

During step 7 (final SP encounter), students take a clinical history from an SP. To prepare students for this encounter, information about the chief complaint is given the day before. After the clinical interview, students perform again a self-assessment but this time they receive feedback from the clinical tutor and from the SP. They also receive information on the history checklist score and the Communication Assessment Scale (CAS) score.

All students enrolled in the program passed in the following high-stake OSCE, contrasting with negative results before the intervention (100% success rate). More specifically, the students enrolled improved their communication skills in the context of a clinical interview with standardized patients.

At the end of the program, students were asked to fulfil an online questionnaire about the sessions. Utility and quality of the sessions were rated on a scale 1-6 with an average score of 5.2. and 4.8, respectively. Students recognize the contribution of the sessions for their result in subsequent exams (average score for this item was 5) and mention that these sessions helped them to perform better in the clinical interview “It helped to systematize the interview and to understand how to order the questions for the differential diagnosis” (S5). The opportunity to “replicate the doctor-patient interaction in the context of training and learning” (S4) is also valued by students. Other positive aspects mentioned by participants were: the “Possibility to view our interviews and of colleagues” (S5);” to train the interview as it will be assessed” (S2); and the fact that faculty and SPs “provide an immediate and comprehensive feedback to the various dimensions of performance” (S4). As an improvement, students suggested that sessions should be more frequent.

Remediation in medical education is a critical process that should be accounted for by every medical school.^6^ The previous consensus emphasize the programmatic view and structure of the remediation programs, using interdisciplinary and multidimensional approaches with clear definition of distinct roles in the remediation process and accountability of the process and its outcomes.^7^ Our results demonstrate that the School of Medicine of Minho’s remediation program, based on such principles, was effective for improving students’ communication and interview skills.

Our approach used a combination of different strategies for an optimal result. In fact, it was shown that combining multiple modalities is more effective than reliance on a single strategy or approach for remediation of medical students.^8^ Interestingly, while every student was engaged in a structured program with diverse modalities of work, our approach also provided the opportunity to draw personalized interventions for each student which contributed to the success of the initiative.

In conclusion, the implementation of a structured remediation program to improve communication skills was successful and well appreciated by students enrolled. The replication of this experiment in other medical schools will allow a better perception of its efficacy and, ultimately by comparison with other remediation strategies, to quantify its advantages.

## Conflict of Interest

The authors declare that they have no conflict of interest.
